# A computational approach for identifying microRNA-target interactions using high-throughput CLIP and PAR-CLIP sequencing

**DOI:** 10.1186/1471-2164-14-S1-S2

**Published:** 2013-01-21

**Authors:** Chih-Hung Chou, Feng-Mao Lin, Min-Te Chou, Sheng-Da Hsu, Tzu-Hao Chang, Shun-Long Weng, Sirjana Shrestha, Chiung-Chih Hsiao, Jui-Hung Hung, Hsien-Da Huang

**Affiliations:** 1 Institute of Bioinformatics and Systems Biology, National Chiao Tung University, Hsin-Chu 300, Taiwan; 2 Graduate Institute of Biomedical Informatics, Taipei Medical University, Taiwan; 3 Department of Biological Science and Technology, National Chiao Tung University, Hsin-Chu 300, Taiwan; 4 Department of Obstetrics and Gynecology, Hsinchu Mackay Memorial Hospital, Hsinchu, Taiwan; 5 Mackay Medicine, Nursing and Management College, Taipei, Taiwan; 6 Department of Medicine, Mackay Medical College, New Taipei City, Taiwan

## Abstract

**Background:**

MicroRNAs (miRNAs) play a critical role in down-regulating gene expression. By coupling with Argonaute family proteins, miRNAs bind to target sites on mRNAs and employ translational repression. A large amount of miRNA-target interactions (MTIs) have been identified by the crosslinking and immunoprecipitation (CLIP) and the photoactivatable-ribonucleoside-enhanced CLIP (PAR-CLIP) along with the next-generation sequencing (NGS). PAR-CLIP shows high efficiency of RNA co-immunoprecipitation, but it also lead to T to C conversion in miRNA-RNA-protein crosslinking regions. This artificial error obviously reduces the mappability of reads. However, a specific tool to analyze CLIP and PAR-CLIP data that takes T to C conversion into account is still in need.

**Results:**

We herein propose the first CLIP and PAR-CLIP sequencing analysis platform specifically for miRNA target analysis, namely miRTarCLIP. From scratch, it automatically removes adaptor sequences from raw reads, filters low quality reads, reverts C to T, aligns reads to 3'UTRs, scans for read clusters, identifies high confidence miRNA target sites, and provides annotations from external databases. With multi-threading techniques and our novel C to T reversion procedure, miRTarCLIP greatly reduces the running time comparing to conventional approaches. In addition, miRTarCLIP serves with a web-based interface to provide better user experiences in browsing and searching targets of interested miRNAs. To demonstrate the superior functionality of miRTarCLIP, we applied miRTarCLIP to two public available CLIP and PAR-CLIP sequencing datasets. miRTarCLIP not only shows comparable results to that of other existing tools in a much faster speed, but also reveals interesting features among these putative target sites. Specifically, we used miRTarCLIP to disclose that T to C conversion within position 1-7 and that within position 8-14 of miRNA target sites are significantly different (*p *value = 0.02), and even more significant when focusing on sites targeted by top 102 highly expressed miRNAs only (*p *value = 0.01). These results comply with previous findings and further suggest that combining miRNA expression and PAR-CLIP data can improve accuracy of the miRNA target prediction.

**Conclusion:**

To sum up, we devised a systematic approach for mining miRNA-target sites from CLIP-seq and PAR-CLIP sequencing data, and integrated the workflow with a graphical web-based browser, which provides a user friendly interface and detailed annotations of MTIs. We also showed through real-life examples that miRTarCLIP is a powerful tool for understanding miRNAs. Our integrated tool can be accessed online freely at http://miRTarCLIP.mbc.nctu.edu.tw.

## Background

MicroRNAs (miRNAs) are about 22-nucletide-length endogenous non-coding RNA molecules that suppress target gene expression. Functional miRNAs typically form RNA-induced silencing complexes (RISCs) that hybridize complementary sequences at 3'-untranslated regions (3' UTRs) of target genes to either degrade mRNA molecules or suppress protein translation [1]. In animals and plants, miRNAs regulate many cellular processes including cell proliferation, differentiation, apoptosis and development [2]. miRNA regulation could be the etiological factor of many diseases including cancer, as well as neurological, and cardiovascular disorders [3]. Biologists have discovered that, on each miRNA, the second to seventh nucleotides (position 2-7) called "seed region" is indispensable for miRNA-target interactions (MTIs) [4]. The seed region in miRNAs should match with the 3' UTR sequence complementarily. So far, the conventionally approaches to verify MTIs such as the reporter assay are still time consuming and incapable of handling the large-scale screening.

Recent works demonstrated that the novel miRNAs, miRNA expression, or MTIs can be uncovered in a large scale by using the next-generation sequencing (NGS) technology. For example, miRDeep [5] predicts the novel miRNAs in NGS data according to a probabilistic model of miRNA biogenesis. Its newest version, miRDeep2 [6], reaches the accuracy around 98.6%-99.9%. Additionally, several tools or web servers were used to identify novel miRNAs or detect miRNA expression levels via NGS such as deepBase [7], Geoseq [8], miRanalyzer [9], SeqBuster [10], mirTools [11], DSAP [12], miRNAkey [13] and miRExpress [14].

Ultraviolet (UV) crosslinking and immunoprecipitation (CLIP) was used to identify specific protein-RNA interaction. Functional miRNA was loaded into Argonate protein and then bound to their target gene to slicing gene expression. Hence the function of Argonate-mRNA-miRNA complex can be verified through CLIP technology. Nowadays, ChIP-seq technology study in protein-DNA interaction by high-throughput sequencing, CLIP-seq technology has been developed to identify protein-RNA interaction by high-throughput sequencing. In 2009, Chi *et al.*[15] pioneered the use of crosslinking and immunoprecipitation (CLIP) method combining with the next-generation sequencing (NGS) technology to discover MTIs in order to obtain Argonaute proteins with mRNA molecules (i.e., targets) in mouse brain. Furthermore, Hafner *et al. *[16] developed a modified CLIP method, namely Photoactivatable-Ribonucleoside-Enhanced Crosslinking and Immunoprecipitation (PAR-CLIP), to enhance the resolution of the original CLIP method. PAR-CLIP enhances protein-RNA crosslinking by introducing photoactivatable ribonucleoside (4-thiouridine, 4SU) into RNAs, makes RNAs sustain in ultra-violet light (UV) with higher energies. Thus, tighter binding was created and results in higher efficiency of RNA co-immunoprecipitation. However it also leads to T to C conversion in the miRNA-RNA-protein crosslinking regions due to the fact that thymine tends to be replaced by 4SU, which could be misidentified as cytosine.

Recently, more and more research groups investigated large-scale MTIs using the CLIP-seq [17-20], and there are several databases, such as CLIPZ [21], starBase [22], doRiNA [23], and TarBase 6.0 [24], compile public available CLIP and PAR-CLIP sequencing datasets and use their in-house software toolkits to analyze the raw data. Among them, only the CLIPZ provides a free web-based analytics environment to the public, and users have to upload their data to the server, which is impractical due to the huge size of the raw sequences and the limited internet bandwidth. Regarding to standalone tools, PARalyzer [25] is the only one that focuses on PAR-CLIP dataset analysis so far, and its execution time is not satisfactory. In other words, there are only two public available tools that are capable of analyzing CLIP and PAR-CLIP sequencing data, and none of them were designed specifically for MTIs.

We herein propose the first CLIP and PAR-CLIP sequencing analysis platform specifically for miRNA target analysis, namely miRTarCLIP. We devised a unique C to T reversion in its workflow to significantly reduce its running time, and included other novel features (see below), which increase miRTarCLIP's functionality. In addition, miRTarCLIP serves with a web-based interface to provide better user experiences in browsing and searching targets of interested miRNAs.

## Results

### An overview of the miRTarCLIP system

**Figure 1:**
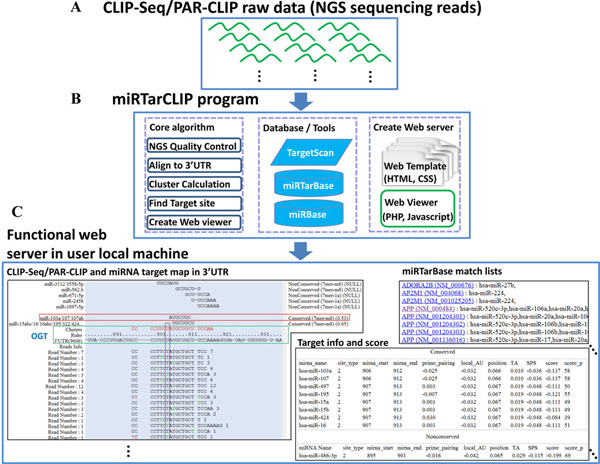
**The system flow of miRTarCLIP**. The miRTarCLIP system flow consists of three parts: (A) preparation of the CLIP/PAR-CLIP sequencing data; (B) loading the raw data into the miRTarCLIP's core algorithms; and (C) presenting the analysis in a web-based browser.

**Figure 2:**
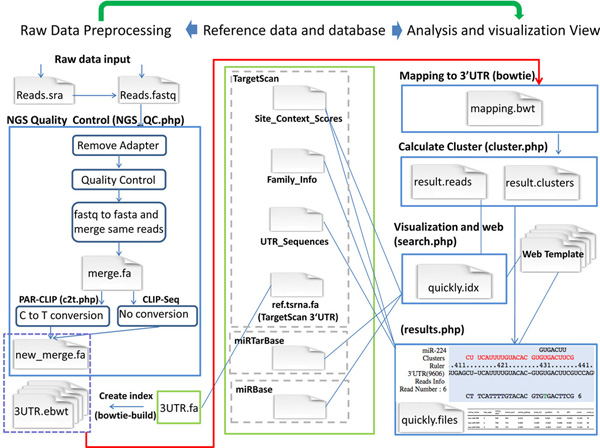
**The miRTarCLIP's core algorithms**. miRTarCLIP automatically removes adaptor sequences from raw reads, filters low quality reads, reverts C to T, aligns reads to 3'UTRs, scans for read clusters, identifies high confidence miRNA target sites, and provides annotations from external databases.

miRTarCLIP consists of six steps (see Methods for details). It automatically removes adaptor sequences from raw reads, filters low quality reads, reverts C to T, aligns reads to 3'UTRs, scans for read clusters, identifies high confidence miRNA target sites, and provides annotations from external databases (Figure 1 and Figure 2). All of the clusters and miRNA target sites and annotations from external databases are automatically presented in a web-based browser created according to a template. The browser also provides a summary table of putative miRNA target sites with scores from TargetScan, target site locations, target gene annotations, and seed region types. In addition, this system takes advantage of the multi-threading technology to enhance the performance.

### The comparison with other CLIP-seq/ PAR-CLIP databases and tools

As mentioned above, several databases and tools, CLIPZ, doRiNA, starBase, and PARalyzer analyze CLIP/PAR-CLIP sequencing datasets. Table 1 lists the major differences among several resources for CLIP/PAR-CLIP data analysis. CLIPZ provides a web service environment for online analysis. PARalyzer [25] is the only stand-alone tool before this work, but it only handles PAR-CLIP data and does not provide a graphical interface. Here, our miRTarCLIP is implemented as a stand-alone tool, which can analyze the new CLIP-seq/PAR-CLIP data on users' local desktops. It provides high-confidence miRNA-target sites with information in detail and presents them in a web-based interface.

**Table 1:** The comparison of miRTarCLIP with other related CLIP/PAR-CLIP sequencing resources

	CLIPZ	doRiNA	StarBase	PARalyzer	miRTarCLIP
**Resource type**	Database/Web tool	Database	Database	Standalone tool	Standalone tool
**Data type**	Both	Both	Both	PAR-CLIP	Both
**Mapping reference**	Genome/transcript	-	-	Genome	23way transcript 3'UTR
**miRNA-target** **interaction**	ElMMo [35]	PicTar [36]	Seed-rule [29]	Seed-rule [29]	TargetScan, [29,30] miRTarBase [31]
**Visualization Browser**	Yes	Yes	Yes	No	Yes

Most uniquely, miRTarCLIP performs a C to T reversion in its workflow for PAR-CLIP dataset, which works along with multithreading techniques to significantly reduce the running time. After mapping reverted reads to 3' UTRs (see Methods), miRTarCLIP clusters reads to search for possible miRNA target sites and uses TargetScan to identify miRNAs that target them. If a candidate miRNA and its target sites had experimental verifications according to miRTarBase, the systems will rank these MTIs on the top of the list in a web-based browser.

### Applying miRTarCLIP to a CLIP-seq dataset

**Figure 3:**
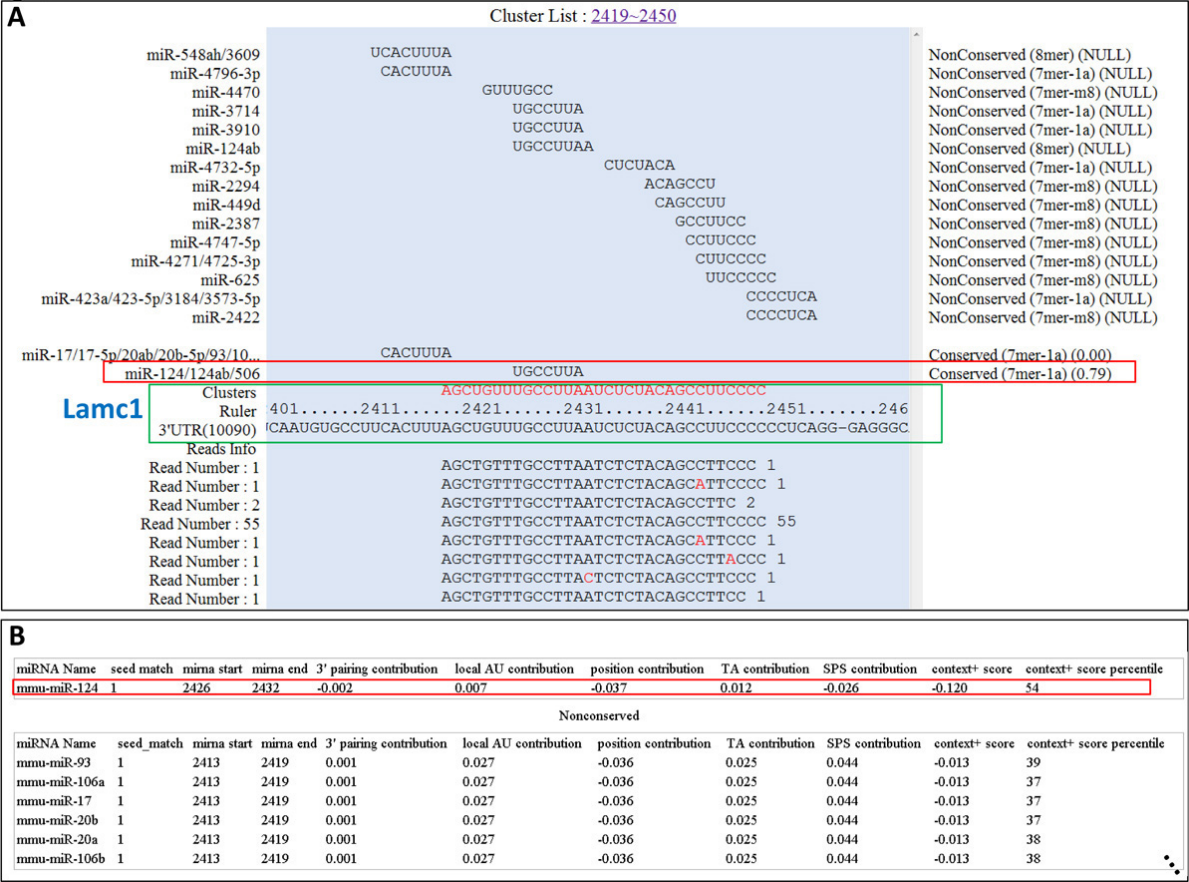
**mmu-miR-124a targets Lamc1 in the Chi et al. CLIP dataset**. (A) A read cluster in Lamc1 3' UTR (position 2418-2449) indicates a candidate AGO-Lamc1-miRNA terney complex (shown in the red sequence within the green rectangle). Above that, a pile of miRNA seed sequences are provided according to the TargetScan. The seed of miR-124 is highlighted in a red box. All the reads of this cluster are aligned underneath the 3' UTR sequence of Lamc1. Red letters in reads are mismatches. (B) Detailed positions and TargetScan context scores of MTIs. According to TargetScan, "seed match" 1 indicates 7mer-A1, which implies perfect match in position 2-7 of the mature miRNA and the nucleotide at position 1 is A in the mRNA target site (defined by TargetScan). Others TargetScan score such as local AU, position, TA, SPS, context+ score, and score percentile are are also defined by TargetScan6.2.

To demonstrate how our system works on CLIP-seq data, it is necessary to apply a dataset for analysis. Additional file 1 shows the web interface of the miRTarCLIP analysing a CLIP-seq data from Chi et al. [15] (BrainA_130_50_fastq). In Additional file 1, Lamc1 and mmu-miR-124 were input in the "Gene Symbol" box and the "miRNA name" box respectively. Lamc1 and miR-124 were chosen because this MTI (miR-124::Lamc1) was experimentally verified by Chi et al. [15]. Figure 3 summarizes the complete annotations and visualization results. In Figure 3A, all possible miRNA-target sites in a read cluster are shown with the miRNA seeds on top. In this case, the read cluster in Lamc1 3' UTR (position 2418-2449) suggests a candidate AGO-Lamc1-miRNA terney complex. According to miRNA expression and the context scores given by TargetScan, miRTarCLIP ranks mmu-miR-124a the most possible miRNA that is involved in this MTI, which is as what we anticipated. Figure 3B gives the locations of miRNA target sites (i.e., miRNA_start and miRNA_end) and the context score from TargetScan.

### Applying miRTarCLIP to a PAR-CLIP sequencing dataset

**Figure 4:**
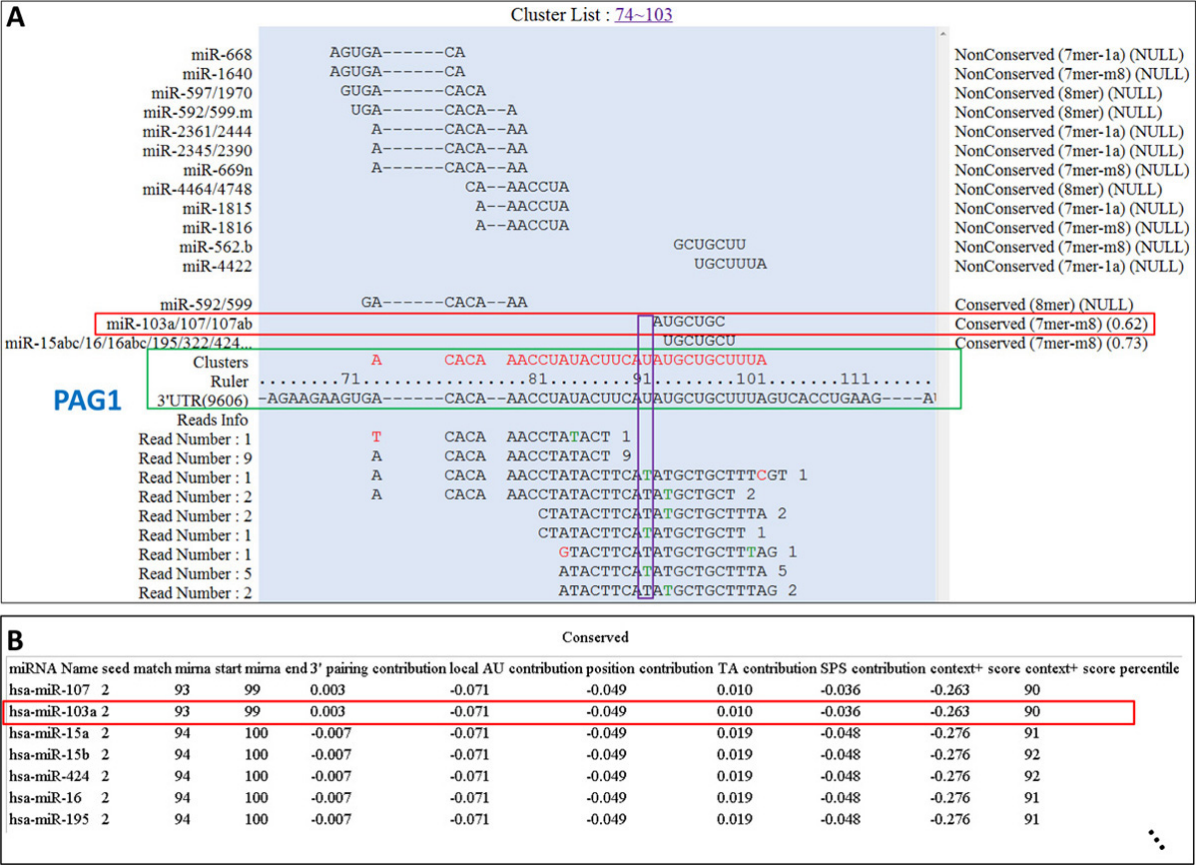
**hsa-miR-103a targets PAG1 in the Hafner et al. PAR-CLIP dataset**. Similar to Figure 3. (A) Specifically to PAR-CLIP dataset, green letters in reads denote the T to C conversion sites. The site with the highest conversion ratio is marked in the purple box. (B) Seed match 2 means that perfect match in position 2-8 of the mature miRNA.

We took the AGO1 PAR-CLIP sequencing dataset (SRR048973) from Hafner et al. [16] as an example. According to Hafner *et al*. [16], miR-103a is a highly expressed miRNA and it targets PAG1. Hafner *et al. *[16] indicated a high T to C conversion at the region between 8^th ^-13^th ^nucleotide in the miRNA target sites. miRTarCLIP identified the same region (position 9 in this case) that contains the most T to C conversion (Figure 4). The system also provides multiple sequence alignments for visualizing conserved target sites among 23 species (Additional file 2). In this case, miR-103a target sites in PAG1 are clearly the conserved ones, but they are less likely targeted in rats because this region is not shown in the alignment (see Additional file 2, 10116 is a taxonomy id of rat). Figure 3 and 4 indicate that miRTarCLIP can produce similar results of the original study and provide novel insights of MTIs.

### The statistic of T to C conversion sites in the Hafner *et al. *[16] PAR-CLIP sequencing dataset

The PAR-CLIP reveals a higher efficiency in RNA co-immunoprecipitation than the regular CLIP. The PAR-CLIP incorporates 4-thiouridine (4SU) into transcripts and applies more energetic UV to enhance the crosslinking between proteins and RNAs, but it also produces artificial T to C conversion. Reads with these errors are difficult to map. Therefore, existing tool, like PARalyzer [25], allows a read to have two mismatches against the reference. However, it dramatically increases the search space and time needed for finding a good match, and in some cases, it could lead to mistaken mappings (see Discussions).

**Figure 5:**
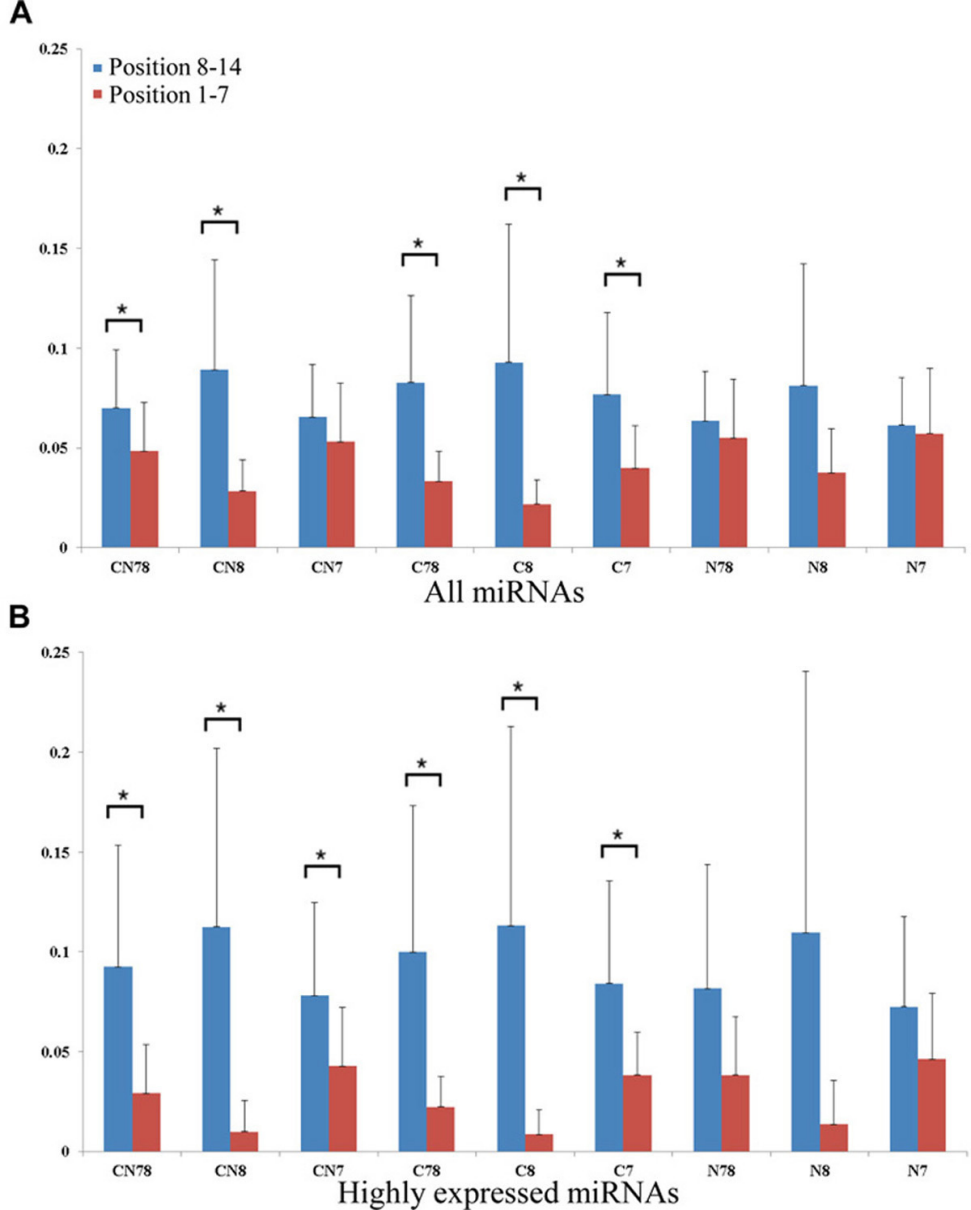
**Comparison of T to C conversion ratio between 8-14 mer and 1-7mer target sites in the Hafner et al. PAR-CLIP sequencing data**. **C**: **C**onserved, **N**: **N**onserved, **7**: **7**mer seed matching, **8**: **8**mer seed matching. For example: The CN78 group consists of miRNA target sites within Conserved, Nonconserved UTRs with both 7mer and 8mer matching. In panel J to R, we used only top 102 expressed miRNAs (from Hefner et al.) to calculate the ratios. (A) All miRNAs (B) top 102 expressed miRNAs. Astric marks indicates significant differnece between position 8-14 and 1-7 (*p *value < 0.05).

Hafner *et al. *[16] and PARalyzer's authors [25] indicated in their works that the ratio of T to C conversion is high in position 8 to 13 of the target sites. The high ratio is considered an evident sign of real miRNA target sites in PAR-CLIP data. To confirm this, we compared the T to C conversion rate within position 1-7 to that within position 8-14 of miRNA target sites, the results indicate that the T to C conversion is significantly different in these two regions (*p *value = 0.02, by one- tailed Student's T test, see Figure 5A, Additional file 3, Table 2). To further understand the association between T to C conversion levels and high-confidence MTIs, we looked for only highly expressed miRNAs and their target sites. The results show that the T to C conversion rates differ in an even higher degree between these regions (*p *value = 0.01. See Figure 5B, Table 2 and Additional file 3). These two results suggest that by incorporating miRNA expression, it is possible to reduce the false positives in finding miRNA targets. The rules of miRNA target prediction usually put constraints on the sequence conservation and miRNA seed regions [4]. Therefore, we also tested whether the conservation and seed regions play a role here. Analytical results indicate that all of the nonconserved seed regions (i.e., N78, N8, N7, see Figure 5, Table 2) and total miRNA/CN7 miRNA-target do not exhibit significant difference (*p *value > 0.05) (Figure 5A, Table 2 and Additional file 3). The results suggest the importance of seeds and conservation. Above results consent with the finding that T to C conversion is located in non-complementary regions of the ternary AGO complex [16,26].

**Table 2:** Comparison of the T to C conversion ratio in different MTI sets

	Total miRNA	Highly expressed miRNA
**CN78**	**0.020**	**0.011**
**CN8**	**0.008**	**0.010**
**CN7**	0.137	**0.047**
**C78**	**0.004**	**0.013**
**C8**	**0.014**	**0.016**
**C7**	**0.014**	**0.030**
**N78**	0.210	0.055
**N8**	0.050	0.055
**N7**	0.357	0.096

MTI sets marked in red have higher T to C conversion ratios in 8th to 14th regions (*p *value < 0.05. See Figure 5)

## Conclusions and discussion

This work develops an integrated approach to analyze CLIP/PAR-CLIP sequencing data in order to identify the miRNA target site. User can study interesting miRNAs or genes/transcripts via a web-based interface. Moreover, the entire source code of miRTarCLIP is freely available on the internet for bioinformatics experts to improve and extend our system.

Comparing with other strategies that allow 2 mismatches in mapping (e.g., PARalyzer [25]), this study introduces a C to T reversion step that tolerates 1 mismatch to reduce the computationally costs and mistaken mapping. Although by doing so (see Methods), we are not free from wrong alignments, but since we only introduce one type of variants (T/C), the chance of getting wrong is only a fraction of what PARalyzer [25] does (allowing one more mismatch actually introduces all pairwise combinations of four nucleotides).

**Figure 6:**
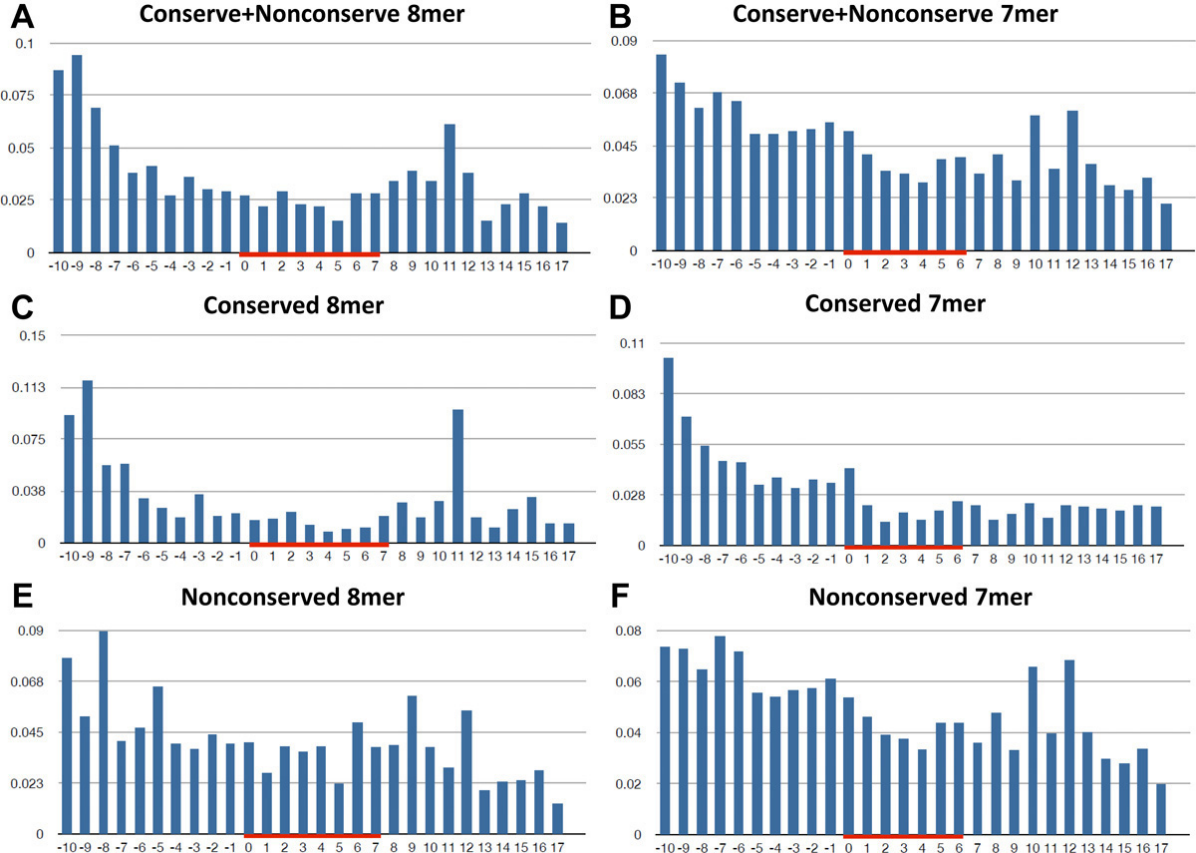
**The distribution of mismatch ratio in the Hafner et al. PAR-CLIP sequencing dataset**. The red lines indicates the miRNA seed regions.

Comparing with the original study (Hafner *et al.*[16]), this study gets the similar results regarding to the statistic of T to C conversion ratio between specific regions. Our analysis further indicates that the regions with high convertion frequency are outside of the seed regions in the conserved targets (Figure 6C). The interesting association between T to C conversion levels and high-confidence MTIs is also investigated using miRTarCLIP. More experimental evidences are needed in the future to clarify the underlying biology.

There are more than 2,000 miRNAs discovered in humans (according to the miRBase version 19), but only less than 300 of them had their MTIs understood by the researchers (according to the miRTarBase version 2.5). The large-scale technologies for discovering MTIs such as CLIP-seq/PAR-CLIP-seq will play a key role in miRNA related studies. We strongly believe miRTarCLIP will be an important resource for the society to reveal more mechanisms of miRNA post-translational regulation.

## Materials and methods

### CLIP-seq and PAR-CLIP datasets

Chi *et al*. [15] recently analyzed MTIs in the mouse brain tissue by high throughput sequencing and CLIP. Hafner *et al.*[16] modified the original CLIP methods by incorporating 4-thiouridine (4SU) into transcripts to increase the efficiency of crosslinking and provide high resolution in protein-RNA binding sites. The raw data of AGO1-AGO4 can be obtained from Gene Expression Omnibus (GEO: GSM545212, GSM545213, GSM545214, GSM545215). The sequencing raw data of these two studies are used in this proposed miRTarCLIP system.

### Information of miRNA and miRNA targets

The miRNA related information, including the accessions and miRNA sequences were obtained from miRBase release 18 [27,28]. microRNA indexes are created to replace miRNA names because of the inconsistency of miRNA naming among different versions of miRBase. The miRNA target prediction and 3' UTR data are obtained from TargetScan release 6.2 [29,30]. The experimentally confirmed MTIs were collected from miRTarBase release 2.5 [31], which was developed previously by our group.

### miRTarCLIP analysis pipeline

Figure 2 illustrates the analysis flow of miRTarCLIP pipeline. The FASTX-Toolkit [32], SRA-Toolkit [33], and bowtie [34] was incorporated into the miRTarCLIP analysis pipeline. The pipeline has six steps: (1) adapter trimming, (2) quality control, (3) C to T reversion, (4) read alignment, (5) cluster analysis, (6) MTI identification analysis. We also take advantage of multi-threading to enhance the performance of the algorithms.

### Step 1: adapter trimming for sequencing reads

This step removes the adapter sequence, if any, at the 3' end of each read. If a trimmed read is shorter than 15 nucleotides or contains any ambiguous nucleotides, the reads are discarded.

### Step 2: quality control of sequencing reads

Following the adapter trimming step, we scan the quality at the tail of each read. The elimination rules are based on the phred quality score. Notably, the nucleotides at the 3' end are removed when their phred scores are lower than 20. Similarly, a reads is discarded if its length less than 15 nucleotides after the tail trimming. Reads with the same sequences are collapsed into one to save the time for mapping duplicates.

### Step 3: cytosine to thymine reversion for PAR-CLIP data

PAR-CLIP technology is implemented by incorporating 4-thiouridone (4SU) to cause thymidine to cytidine transition in the RNA binding protein sites on transcripts. For each cytidine in a read, this step will create a new read with that C converted to T. For instance, a read sequence, AATG**C**T**C**AATGG**C**GA, will be converted to AATG**T**T**C**AATGG**C**GA, AATG**C**T**T**AATGG**C**GA, and AATG**C**T**C**AATGG**T**GA. All four sequences (i.e., one original read and three converted reads) are used to align against the references.

### Step 4: aligning sequencing reads against reference sequences

miRNAs target mRNAs at 3' UTRs, so instead of aligning reads to the entire genome, we use exclusively the 3' UTR sequences from TargetScan. The reads are mapped with at most one mismatch. Other tools, like PARalyzer [25] uses two mismatches to address the T to C conversion issue in PAR-CLIP dataset. However, allowing two mismatches in mapping (e.g., using **bowtie) **is very time consuming and error-prone. To resolve this problem, a better strategy is to revert C back to T in reads (as described in Step 3), and align them to the references with at most one mismatch, in which reduces the computational costs. We have tested our results with published PAR-CLIP data from Hafner *et al.*[16] (SRX020783). We validated the fact that our C to T reversion combining with one mismatch mapping tolerance in bowtie is more efficient than doing mapping directly by allowing 2 mismatches. The result shows that we reduced the computation time by two folds and generated 0.64 folds output despite our C to T reversion introduced 7 folds of extra input (Table 3).

**Table 3:** Comparison of computational time and bowtie mapping

	miRTarCLIP	PARalyzer	Comparison
**Times **(sec)			
C to T program	11.7	-	
Bowtie	135	301.7	
**Total**	**146.7**	**301.7**	**2 folds**
**Bowtie input and output**			
Input reads	6429483	919698	**7 folds**
Output reports	10785713	16895608	**0.64 folds**

# Dataset: PAR-CLIP data from Hafner et. al. (SRX020783).

# Bowtie parameter: one mismatch in miRTarCLIP and two mismatch in PARalyzer.

# System: Linux x64, Intel(R) Xeon(R) CPU E5620 @ 2.40GHz, 16G RAM.

### Step 5: cluster searching and analysis

These reads are clustered based on their minimum overlap between each other, at least 20% of the reads in a cluster should have the T to C conversion; the minimum number of reads in a cluster is five reads. In the PAR-CLIP dataset, a cluster reads should contain at least 20% of the T to C conversion. Whether the cluster sequence is a possible target site is confirmed using the miRNA seed region sequences extracted from miRBase.

### Step 6: miRNA-target interaction (MTI) analysis

The clustering results are used to search for possible miRNA target sites by TargetScan. If a candidate target site is experimentally validated according to miRTarBase, the system will display it on the top. Other candidates will be ranked according to the context scores assigned by TargetScan.

## Availability and requirements

miRTarCLIP software was implemented by PHP programming language and integrated FASTX-Toolkit, SRA-Toolkit and a bowtie program written in C++ programming language. The software can be executed in 32 or 64 bit Linux machine. The software and case study results can be accessed online at http://miRTarCLIP.mbc.nctu.edu.tw.

## Competing interests

The authors declare that they have no competing interests.

## Authors' contributions

CHC carried out all experimental concepts, wrote part of the program and the manuscript. JHH organized the study, and write the manuscript. FML carried out some experimental concepts and assisted in the design of the study. MTC assisted in the design of the study and programming. SDH, THC, SLW, SS, and CCH assisted in the design of the study. HDH managed the study in the initial model, and assisted write and revise the manuscript. All authors read and approved the final manuscript.

## Declaration

The authors approve the submission of this paper to *BMC Genomics* for publication. The payment of a publishing charge to BioMed Central for this article was supported by National Science Council of the Republic of China, No. NSC 101-2311-B-009-003-MY3 and NSC 100-2627-B-009-002. This publishing charge was supported in part by the UST-UCSD International Center of Excellence in Advanced Bio-engineering sponsored by the Taiwan National Science Council I-RiCE Program under Grant Number: NSC 101-2911-I-009-101, and Veterans General Hospitals and University System of Taiwan (VGHUST) Joint Research Program under Grant Number: VGHUST101-G5-1-1. This publishing charge was also partially supported by MOE ATU.

This article has been published as part of *BMC Genomics *Volume 14 Supplement 1, 2013: Selected articles from the Eleventh Asia Pacific Bioinformatics Conference (APBC 2013): Genomics. The full contents of the supplement are available online at http://www.biomedcentral.com/bmcgenomics/supplements/14/S1.

## Supplementary Material

Additional file 1 **The web-based browser interface of the miRTarCLIP system**. Click here for file

Additional file 2 **The multiple species sequence alignment viewer**. Click here for file

Additional file 3 **The distribution of T to C conversion ratio around target sites in the Hafner et al. PAR-CLIP sequencing data**. Click here for file
