# Supplementary material for: A computational approach for identifying microRNA-target interactions using high-throughput CLIP and PAR-CLIP sequencing
Source: BMC Genomics. 2013 Jan 21;14(Suppl 1):S2. doi: 10.1186/1471-2164-14-S1-S2 (PMC3549799; doi:10.1186/1471-2164-14-S1-S2)

**Additional file 1**. **The web-based browser interface of the miRTarCLIP system.** Users can search for MTIs by miRNA names, target genes’ Entrez symbol and accession numbers, or directly browse by the Entrez gene symbols. For example, Lamc1 was input in “Gene Symbol” box and mmu-miR-124 in “miRNA name” box. The results were shown in the bottom-right panel.


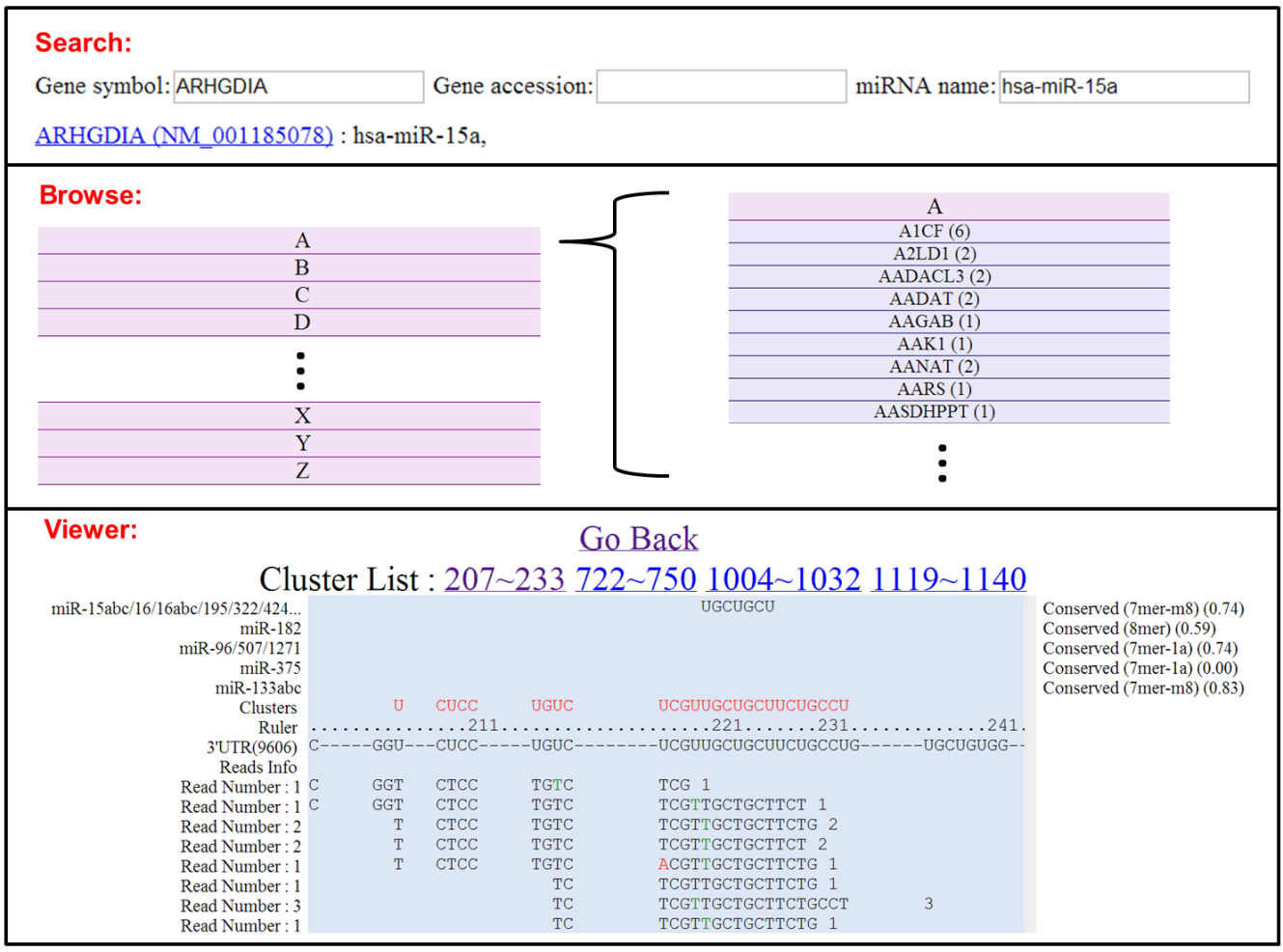

Supplement: Additional file 1 — The web-based browser interface of the miRTarCLIP system. [file 1471-2164-14-S1-S2-S1.doc]
