# Supplementary material for: A computational approach for identifying microRNA-target interactions using high-throughput CLIP and PAR-CLIP sequencing
Source: BMC Genomics. 2013 Jan 21;14(Suppl 1):S2. doi: 10.1186/1471-2164-14-S1-S2 (PMC3549799; doi:10.1186/1471-2164-14-S1-S2)

**Additional file 2. The multiple species sequence alignment viewer**.

The system provides a multiple species sequence alignment for mining conserved target sites among species.


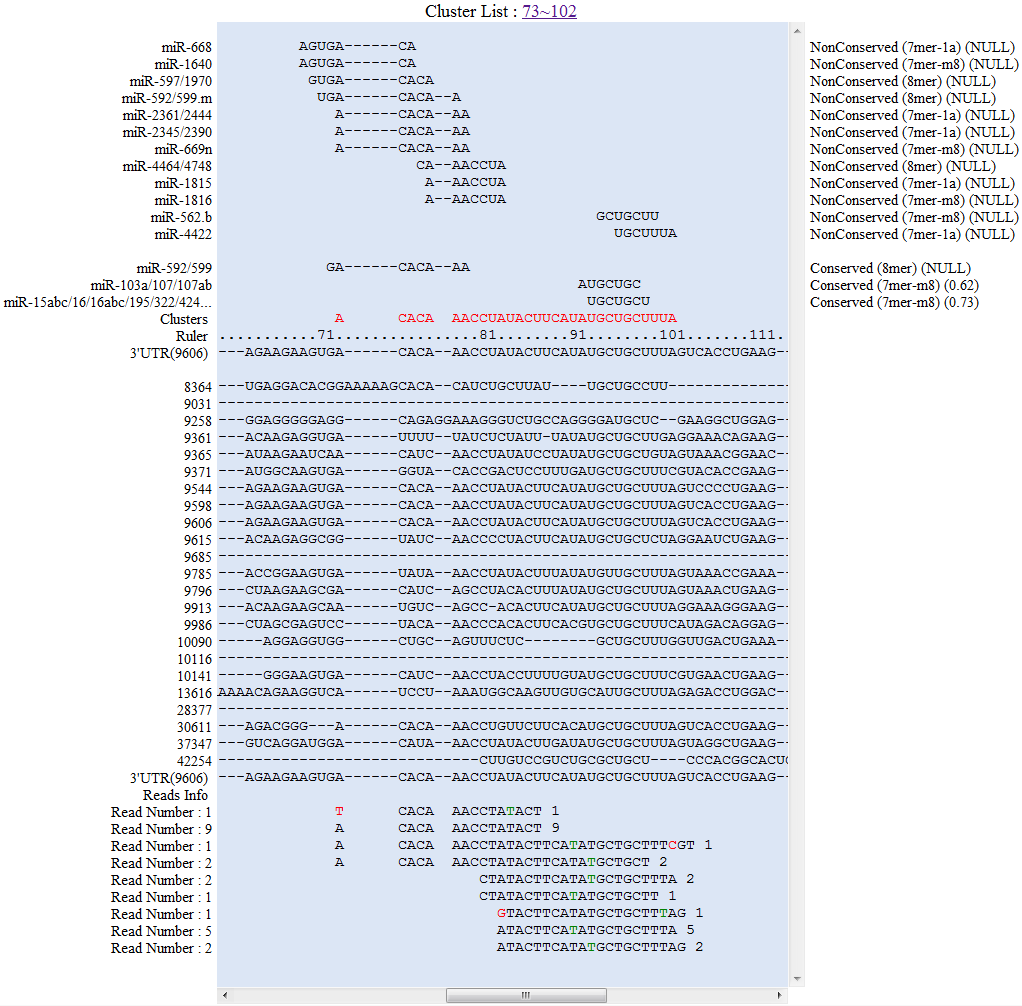

Supplement: Additional file 2 — The multiple species sequence alignment viewer. [file 1471-2164-14-S1-S2-S2.doc]
