# Supplementary material for: A computational approach for identifying microRNA-target interactions using high-throughput CLIP and PAR-CLIP sequencing
Source: BMC Genomics. 2013 Jan 21;14(Suppl 1):S2. doi: 10.1186/1471-2164-14-S1-S2 (PMC3549799; doi:10.1186/1471-2164-14-S1-S2)
Supplement: Additional file 3 — The distribution of T to C conversion ratio around target sites in the Hafner et al. PAR-CLIP sequencing data. [file 1471-2164-14-S1-S2-S3.doc]

**Additional file 3. The distribution of T to C conversion ratio around target sites in the Hafner et al. PAR-CLIP sequencing data. C**: **C**onserved, **N**: **N**onserved, **7**: **7**mer seed matching, **8**: **8**mer seed matching. For example: The CN78 group consists of miRNA target sites within Conserved, Nonconserved UTRs with both 7mer and 8mer matching. In panel J to R, we used only top 102 expressed miRNAs (from Hefner et al.) to calculate the ratios.
